# Deciphering Sex-Specific Genetic Architectures Using Local Bayesian Regressions

**DOI:** 10.1534/genetics.120.303120

**Published:** 2020-03-18

**Authors:** Scott A. Funkhouser, Ana I. Vazquez, Juan P. Steibel, Catherine W. Ernst, Gustavo de los Campos

**Affiliations:** * Institute for Behavioral Genetics, The University of Colorado, Boulder, Colorado 80309; † Genetics Graduate Program, Michigan State University, East Lansing, Michigan 48824; ‡ Departments of Epidemiology and Biostatistics and Statistics and Probability, Institute for Quantitative Health Science and Engineering, Michigan State University, East Lansing, Michigan, 48824; § Department of Animal Science, Michigan State University, East Lansing, Michigan, 48824

**Keywords:** Bayesian methods, gene-by-sex interactions, GWAS

## Abstract

Many complex human traits exhibit differences between sexes. While numerous factors likely contribute to this phenomenon, growing evidence from genome-wide studies suggest a partial explanation: that males and females from the same population possess differing genetic architectures. Despite this, mapping gene-by-sex (G×S) interactions remains a challenge likely because the magnitude of such an interaction is typically and exceedingly small; traditional genome-wide association techniques may be underpowered to detect such events, due partly to the burden of multiple test correction. Here, we developed a local Bayesian regression (LBR) method to estimate sex-specific SNP marker effects after fully accounting for local linkage-disequilibrium (LD) patterns. This enabled us to infer sex-specific effects and G×S interactions either at the single SNP level, or by aggregating the effects of multiple SNPs to make inferences at the level of small LD-based regions. Using simulations in which there was imperfect LD between SNPs and causal variants, we showed that aggregating sex-specific marker effects with LBR provides improved power and resolution to detect G×S interactions over traditional single-SNP-based tests. When using LBR to analyze traits from the UK Biobank, we detected a relatively large G×S interaction impacting bone mineral density within *ABO*, and replicated many previously detected large-magnitude G×S interactions impacting waist-to-hip ratio. We also discovered many new G×S interactions impacting such traits as height and body mass index (BMI) within regions of the genome where both male- and female-specific effects explain a small proportion of phenotypic variance (R^2^ < 1 × 10^−4^), but are enriched in known expression quantitative trait loci.

SEX differences are widespread in nature, and observed readily among many human traits and diseases. For quantitative traits, sex may affect the distribution of phenotypes at various levels, including mean-differences between genetic males and genetic females (hereafter referred to as males and females, respectively) as well as differences in variance. Sex differences are likely due to myriad factors, including differential environmental exposures and unequal gene dosages for sex-linked genes, as well as sex-heterogeneity in the architecture of genetic effects at one or more autosomal loci [*i.e.*, gene-by-sex (G×S) interactions]. In this way, sex provides two well-defined conditions in which allele frequencies and linkage disequilibrium (LD) patterns are equivalent, but nevertheless genetic effects of one or many autosomal loci may differ.

Evidence for different genetic architectures between sexes among human populations is largely supported by genome-wide parameters (Weiss *et al.* 2006; Zillikens *et al.* 2008; Yang *et al.* 2015; Rawlik *et al.* 2016) including unequal within-sex heritabilities (*h*^2^_male_ *≠ h*^2^_female_) and between-sex genetic correlations *r*_g_ < 1; the former suggests that the proportion of phenotypic variance explained by genetic factors varies between sexes, while the latter suggests genetic effects are disproportional between sexes (Lynch 1998). Although many traits seem to have a between-sex genetic correlation that is evidentially <1, genome-wide association (GWA) studies intended to map G×S interactions have struggled to pinpoint such loci (Liu *et al.* 2012; Hoffmann *et al.* 2017). Based on this dichotomy, G×S interactions presumably exist for many traits, but the magnitude of a typical G×S interaction is suspected to be exceedingly small, explaining why such events commonly elude detection, particularly after multiple test correction. However, just as numerous small effect causal loci accumulate to affect phenotypic variance, small G×S interactions may accumulate to influence both sex differences and phenotypic variance.

Most GWA studies utilize single-marker regression (SMR), in which the phenotype is regressed upon allele content one SNP at a time, thereby obtaining marginal SNP effect size estimates that do not fully account for LD patterns. In contrast, whole-genome regression methods, in which the phenotype is regressed upon all SNPs across the genome concurrently, fully account for multi-locus LD. These methods are increasingly being used as a one-stop solution to estimate conditional (with respect to other SNPs) effect sizes of SNP markers and to provide genome-wide estimates including genomic heritability (Meuwissen *et al.* 2001; Yang *et al.* 2010; de Los Campos *et al.* 2015a) and between-sex genetic correlations (Zillikens *et al.* 2008; Yang *et al.* 2015; Rawlik *et al.* 2016). By estimating conditional SNP effect sizes, the goal across many studies is to select SNPs with nonzero effects and to build a model for predicting polygenic scores (de los Campos *et al.* 2010, 2013; Lello *et al.* 2018). Other works have directly illustrated the use of whole-genome regression methods for GWAS (Yi *et al.* 2003; Ayers and Cordell 2010; Guan and Stephens 2011; Zeng *et al.* 2012; Fernando *et al.* 2017). Whole-genome regressions are computationally challenging to use with biobank-level data; however, recent work suggests relatively accurate genomic prediction and SNP effect estimation can be achieved simply by accounting for local (as opposed to global) LD patterns (Vilhjálmsson *et al.* 2015).

Building on the idea of utilizing conditional SNP marker effects, here we developed local Bayesian regressions (LBR) in which the phenotype is regressed upon multiple SNPs spanning multiple LD blocks (thereby accounting for local LD patterns) to study sex differences in complex traits from the UK Biobank. The LBR model uses random-effect SNP-by-sex interactions (de Los Campos *et al.* 2015b; Veturi *et al.* 2019) that decompose conditional SNP effects into three components: (i) one shared across sexes, (ii) a male-specific deviation from the shared component, and (iii) a female-specific deviation from the shared component. Using samples from the posterior distribution of conditional SNP effects, we developed methods to infer sex-specific effects and G×S interactions at the single SNP level and by aggregating SNP effects within small LD-based regions, offering multiple perspectives to study sex-specific genetic architectures.

In this study, we have utilized genotypes for 607,497 autosomal SNPs from ∼259,000 distantly related Caucasians from the UK Biobank for assessing the performance of LBR in analyzing simulated and real complex traits including height, body mass index (BMI), waist-to-hip ratio (WHR), and heel bone mineral density (BMD). Using simulations, we showed that (i) for inferences of G×S interactions, LBR offers higher power with lower false discovery rate (FDR) than methods based on marginal effects (aka single-marker regression), and (ii) under imperfect LD between SNPs and causal variants (*i.e.*, when causal variants are not genotyped), aggregating SNP effects within small LD-based regions offers higher power than methods based on testing individual SNPs.

The traits analyzed in this study span a range of genome-wide metrics and G×S plausibility, from height and BMI, for which previous studies indicate males and females possess very similar genetic architectures (Yang *et al.* 2015), to WHR, a trait with well-documented G×S interactions (Heid *et al.* 2010; Randall *et al.* 2013; Shungin *et al.* 2015; Winkler *et al.* 2015), and BMD, for which G×S interactions are thought to exist but have eluded detection (Karasik and Ferrari 2008). LBR provided evidence of G×S interactions impacting height, BMI, and BMD at regions of the genome where sex-specific genetic effects are relatively small; however, such regions are enriched in known eQTL. For WHR, LBR replicated many large-magnitude G×S interactions previously discovered using single-marker regression, but also located novel G×S interactions near such genes as the estrogen receptor *ESR1*.

## Materials and Methods

### Genotype and phenotype data

Genotyped SNPs from the custom UK Biobank Axiom Array (http://www.ukbiobank.ac.uk/scientists-3/uk-biobank-axiom-array/) were used in all analysis. Prior to analysis, all phenotypes were precorrected for sex, age, batch, genotyping center, and the first five genomic principal components. More information on genotypes and phenotypes can be found in the Supplemental Methods.

### Overview of the LBR model, inference methods, and implementation

Here, we present brief details of the LBR model and implementation, with more details found in the Supplemental Methods. An example of how to fit LBR and perform postprocessing of posterior samples of model parameters is available at: https://github.com/funkhou9/LBR-sex-interactions.

To study sex differences, we regressed male and female phenotypes ($y_{\text{m}}$ and $y_{\text{f}}$) on male and female genotypes ($X_{\text{m}}$ and $X_{\text{f}}$) using a SNP-by-sex interaction model of the form

$$\begin{array}{c} \left[\begin{array}{c} y_{\text{m}}\\ y_{\text{f}} \end{array}\right]=\left[\begin{array}{c} 1\mu_{\text{m}}\\ 1\mu_{\text{f}} \end{array}\right]+\left[\begin{array}{c} X_{\text{m}}\\ X_{\text{f}} \end{array}\right]b_{0}+\left[\begin{array}{c} X_{\text{m}}\\ 0 \end{array}\right]b_{\text{m}}+\left[\begin{array}{c} 0\\ X_{\text{f}} \end{array}\right]b_{\text{f}}+\left[\begin{array}{c} \varepsilon_{\text{m}}\\ \varepsilon_{\text{f}} \end{array}\right]. \end{array}$$ (1)

Above, $\mu_{\text{m}}$ and $\mu_{\text{f}}$ are male and female intercepts, $b_{0}=\left\{b_{0_{j}}\right\}$ (*j* = 1, …, *p*) is a vector of main effects, $b_{\text{m}}=\left\{b_{{\text{m}}_{j}}\right\}$ and $b_{\text{f}}=\left\{b_{{\text{f}}_{j}}\right\}$ are male and female interactions, respectively, and $\varepsilon_{\text{m}}=\left\{\varepsilon_{{\text{m}}_{i}}\right\}$ and $\varepsilon_{\text{f}}=\left\{\varepsilon_{{\text{f}}_{i}}\right\}$ are male and female errors that were assumed to follow normal distributions with zero mean and sex-specific variances. Female-specific and male-specific SNP effects are defined as $\beta_{{\text{f}}_{j}}=b_{0_{j}}+b_{{\text{f}}_{j}}$ and $\beta_{{\text{m}}_{j}}=b_{0_{j}}+b_{{\text{m}}_{j}},$ respectively.

#### Prior assumptions:

For SNP effects, we adopted priors from the spike-slab family with a point of mass at zero and a Gaussian slab (Habier *et al.* 2011); specifically, $Pr\left(b_{k_{j}}\right)=\pi_{k}N\left(0,\sigma_{b_{k}}^{2}\right)+\left(1-\pi_{k}\right)1\left(b_{k_{j}}=0\right),$ where k=0,f or m. Here, $\pi_{k}$ and $\sigma_{b_{k}}^{2}$ are hyper-parameters representing the proportion of nonzero effects and the variance of the slab; these hyper-parameters were treated as unknown and given their own hyper-priors.

#### Local-regression:

**Figure 1:**
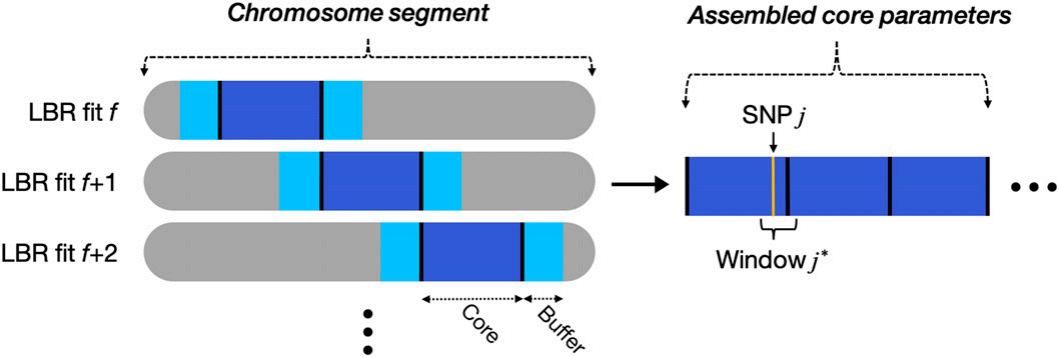
Strategy for implementing local Bayesian regressions genome-wide. The phenotype is regressed upon multiple sequential SNPs using a sliding window approach. The core region contained 1500 SNPs (roughly 8 Mb, on average), and each buffer region contained 250 SNPs (roughly 1 Mb, on average). Core parameters (posterior samples) are stitched together, then sex-specific effects and G×S interactions are inferred at the level of SNP *j* and window j*.

Implementing the above model with whole-genome SNPs (*p* ∼600K) and very large sample size (*n ∼*300K) is computationally extremely challenging. However, LD in homogeneous unstructured human populations spans over relatively short regions (*R*^2^ between allele dosages typically vanishes within 1–2 Mb; Supplemental Material, Figure S1). Therefore, we applied LBR to long, overlapping chromosome segments (Figure 1). Specifically, we divided the genome into “core” segments containing 1500 contiguous SNPs (roughly 8 Mb, on average), then applied the regression in Equation 1 to SNPs in the core segment plus 250 SNPs (*i.e.*, roughly 1 Mb) in each flanking region, which were added to account for LD between SNPs at the edge of each core segment with SNPs in neighboring segments.

#### Inferences:

We used the BGLR (Pérez and De Los Campos 2014) software to draw samples from the posterior distribution of the model parameters, and used these samples to make inference about individual SNP effects including: (i) the posterior probability that the *j*^th^ SNP has a nonzero effect in males $\left({\text{PPM}}_{{\text{SNP}}_{j}}\right)$ and females $\left({\text{PPF}}_{{\text{SNP}}_{j}}\right),$ and (ii) the posterior probability that the female and male effects are different $\left({\text{PPDiff}}_{{\text{SNP}}_{j}}\right).$

In regions involving multiple SNPs in strong LD, inferences at the individual-SNP level may be questionable. Therefore, we borrowed upon previous work by Fernando *et al.* (2017), enabling us to aggregate multiple sex-specific SNP effects within relatively small regions using “window variances.” For each SNP *j* we defined a window j* around the SNP based on local LD patterns. We then defined the male-specific and female-specific window variances as $\sigma_{g_{\text{m}}{}_{j^{*}}}^{2}=var\left(X_{j^{*}}\beta_{{\text{m}}_{j^{*}}}\right)$ and $\sigma_{g_{\text{f}}{}_{j^{*}}}^{2}=var\left(X_{j^{*}}\beta_{{\text{f}}_{j^{*}}}\right),$ respectively. Here, $X_{j^{*}}$ represent genotypes at SNPs within the j* window, and var() is the sample variance operator. Prior to model fitting, the phenotype is scaled across sexes; thus, sex-specific window variances may be interpreted as the proportion of total phenotypic variance explained by sex-specific SNP effects. From samples of sex-specific window variances, we computed the posterior probability of (i) nonzero male-specific window variance $\left({\text{PPM}}_{\sigma_{g_{j^{*}}}^{2}}\right),$ (ii) nonzero female-specific window variance $\left({\text{PPF}}_{\sigma_{g_{j^{*}}}^{2}}\right),$ and (iii) sex difference in window variances (denoted as ${\text{PPDiff}}_{\sigma_{g_{j^{*}}}^{2}}$).

### Overview of simulations

We used simulations to assess the power and FDR of LBR, and to compare them with that of standard single-marker-regression (SMR). Traits were simulated using SNP genotypes from the UK Biobank (119,190 males and 139,738 females, all distantly related Caucasians), thus providing realistic LD patterns. We simulated a highly complex trait with one causal variant per ∼2 Mb, which, on average, explained a proportion of the phenotypic variance equal to 3.3 × 10^−4^. Our simulations used a total of 60,000 genotyped SNPs (∼one-tenth of the genome, consisting of 6000 consecutive SNPs taken from 10 different chromosomes) and 150 causal variants; on the complete human genome “scale” this corresponds to a trait with 1500 causal variants and a heritability of 0.5 (see Supplemental Methods for further details). Of the causal variants, 40% (a total of 60 SNPs) had differing sex-specific effects and the remaining 60% (90 SNPs) had effects that were the same in males and females. Estimates of power and FDR were based on 30 Monte Carlo (MC) replicates.

### Data availability

The authors state that all data necessary for confirming the conclusions presented in the article are represented fully within the article. Genotype and phenotype data from the UK Biobank are available to all researchers upon application at http://www.ukbiobank.ac.uk/register-apply/. The script used to simulate phenotypes is available at https://github.com/funkhou9/LBR-sex-interactions. For eQTL enrichment analysis, single-tissue *cis*-eQTL data (significant variant-gene associations based on permutations) from GTEx V7 was downloaded from https://gtexportal.org/home/datasets.

Supplemental Methods, Figure S1 through S5, and Table S1 through S4 are available at Figshare at https://doi.org/10.25386/genetics.11900247.

## Results

### LBRs offer improved power with lower FDRs

#### Power and FDR when causal variants are genotyped:

**Figure 2:**
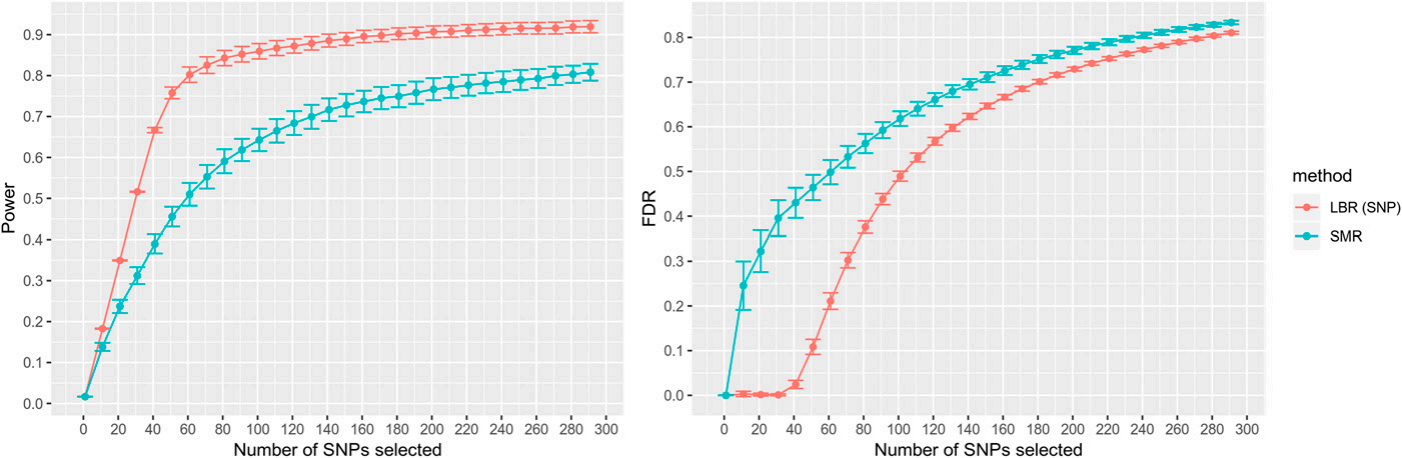
Estimated power and false discovery rate for discovering observed SNPs with G×S interactions. Shown as a function of the number of SNPs selected. Each point represents a sample average and error bars represent 95% confidence intervals, each derived using 30 Monte Carlo replicates. LBR (SNP): local Bayesian regression, utilizing ${\text{PPDiff}}_{\text{SNP}}.$ SMR: single-marker regression, utilizing *P value*-diff.

First, we analyzed the simulated phenotypes using all SNPs (including all 150 causal SNPs). Initially interested in inferring G×S interactions, we ranked SNPs based on the ${\text{PPDiff}}_{\text{SNP}}$ metric of LBR and on SMR’s *P*-value for sex difference (*P value*-diff, see Supplemental Methods) and used the two ranks to estimate power and FDR as a function of the number of SNPs selected (Figure 2). We observed little variation in power and false discoveries across MC replicates (Table S2); this was expected because each MC replicate involved 60,000 loci, 150 of which had causal effects. LBR showed consistently higher power (achieving a power of ∼75% when selecting the top 50 SNPs with highest ${\text{PPDiff}}_{\text{SNP}}$) and lower FDR than SMR. The FDR of LBR was very low when selecting the top 50 SNPs with highest ${\text{PPDiff}}_{\text{SNP}}$ and exhibited a very sharp phase-transition with fast increase in FDR thereafter.

We also compared the two methods based on arbitrary, albeit commonly used, mapping thresholds for SMR and LBR. At ${\text{PPDiff}}_{\text{SNP}}\geq 0.95$ [a posterior probability threshold used in GWAS to minimize the local false discovery rate (Efron 2008)], LBR selected an average (across simulation replicates) of 38.33 SNPs with an estimated power of 0.634 and estimated FDR of 0.007. Conversely, at *P value*-diff ≤ 5 × 10^−8^ [a *P*-value threshold routinely used in human GWA studies, based on the number of approximately independent SNPs in the human genome (International HapMap Consortium *et al.* 2007)] SMR selected an average of 50.7 SNPs with an estimated power of 0.436 and estimated FDR of 0.451. Altogether, these results suggest that for G×S discovery, LBR offers higher power and lower FDR than SMR—the method most widely used in GWA studies—at least when G×S interactions are observed.

When trying to map SNPs that had effect in at least one sex, we used ${\text{PP}}_{{\text{SNP}}_{j}}=\text{max}\left[{\text{PPM}}_{{\text{SNP}}_{j}},\;{\text{PPF}}_{{\text{SNP}}_{j}}\right]$ and *P*-values from an *F*-test (see Supplemental Methods) as metrics for LBR and SMR methods, respectively. Again, LBR showed higher power with lower FDR than a standard SMR *P*-value (Figure S2). At traditional mapping thresholds, LBR and SMR had similar power but LBR achieved that power with much lower FDR; at ${\text{PP}}_{{\text{SNP}}_{j}}\geq 0.95$, the average number of SNPs selected was 120.83 with an estimated power of 0.799 and estimated FDR of 0.009, whereas at *P*-value ≤ 5 × 10^−8^, the number of SNPs selected was 374.56 with an estimated power of 0.794 and FDR of 0.66.

#### Power and FDR when causal variants are masked:

**Figure 3:**
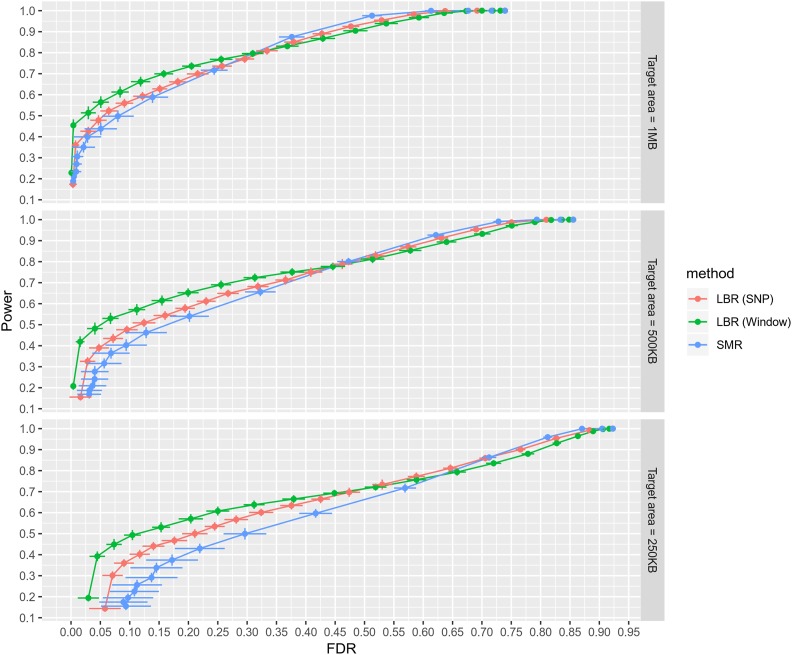
Power *vs.* false discovery rate for discovering genomic regions containing masked G×S interactions. Here, power is defined as the expected proportion of G×S interactions that are being tagged by at least one selected SNP *j* or window j*. False discovery rate is defined as the expected proportion of selected SNPs or windows that are not tagging any G×S interactions. Each point is an estimate, and error bars for both axes represent 95% confidence intervals. Point estimates and intervals were derived using 30 Monte Carlo replicates. Each facet corresponds to a different “target area,” a fixed width around each G×S interaction that defines the set of SNPs effectively tagging it. LBR (SNP): uses the ${\text{PPDiff}}_{\text{SNP}}$ metric spanning 1-0. LBR (Window): uses the ${\text{PPDiff}}_{\sigma_{g}^{2}}$ metric spanning 1-0. SMR: uses the *P value*-diff metric spanning (on the –log_10_ scale) 8-0.

In a second round of analyses, we removed all causal variants from the panel of SNPs used in the analysis to represent a situation where causal variants are not observed, and genotyped SNPs are tagging causal variants at varying degrees. We initially assessed the relative performance of LBR to infer segments harboring G×S interactions. Power and FDR were assessed at several resolutions: 1 Mb, 500 kb, and 250 kb regions around each causal variant. At each resolution, a discovery was considered true if the finding laid within a segment harboring a G×S causal variant. In this scenario, we again observed that LBR had small variability in power and false discoveries between MC replicates (Table S3). Power and FDR were computed at different thresholds (using ${\text{PPDiff}}_{\text{SNP}}$ and ${\text{PPDiff}}_{\sigma_{g}^{2}}$ for LBR and *P value*-diff for SMR; Figure 3). When using a 1 Mb target area—such that correct G×S discoveries must be within 500 kb on either side of a true G×S event—${\text{PPDiff}}_{\sigma_{g}^{2}}$ thresholds (LBR’s window-based metric) provided highest power within an FDR range of 0–0.3; thereafter, SMR provided slightly higher power. As expected, when removing causal variants, power was estimated to be much lower than when causal variants were observed; at ${\text{PPDiff}}_{\sigma_{g}^{2}}\geq 0.95,$ the estimated power and FDR were 0.454 and 0.004, respectively, while at *P value*-diff ≤ 5 × 10^−8^, estimated power and FDR were 0.22 and 0.006. As seen in Figure 3, when considering a finer resolution (500 kb and 250 kb) the performance of both LBR-based approaches was more robust than that of SMR. Altogether this indicates that for the discovery and mapping of unobserved G×S interactions, LBR’s window-based metric provides higher power with equivalent FDR and finer resolution than single-marker regression methods.

To infer segments containing causal variants that affect at least one sex, we again used LBR to decide whether either sex-specific effect was nonzero at the level of individual SNPs or windows. Using a 1-MB target area, LBR’s window-based metrics provided the highest power within an FDR range of 0–0.025. When decreasing the target area, LBR provided the highest power over larger FDR ranges (Figure S3).

### For real human traits, many novel G×S interactions showed relatively small sex-specific effects

We analyzed four complex human traits (height, BMI, BMD, and WHR) measured among ∼259,000 distantly related Caucasians from the UK Biobank (∼119,000 males and ∼140,000 females). For each trait, we fit the LBR model (Equation 1) across the entire autosome consisting of 607,497 genotyped SNPs using 417 overlapping segments (Figure 1) and obtained evidence of G×S interactions at the level of SNP *j* and window j*.

**Figure 4:**
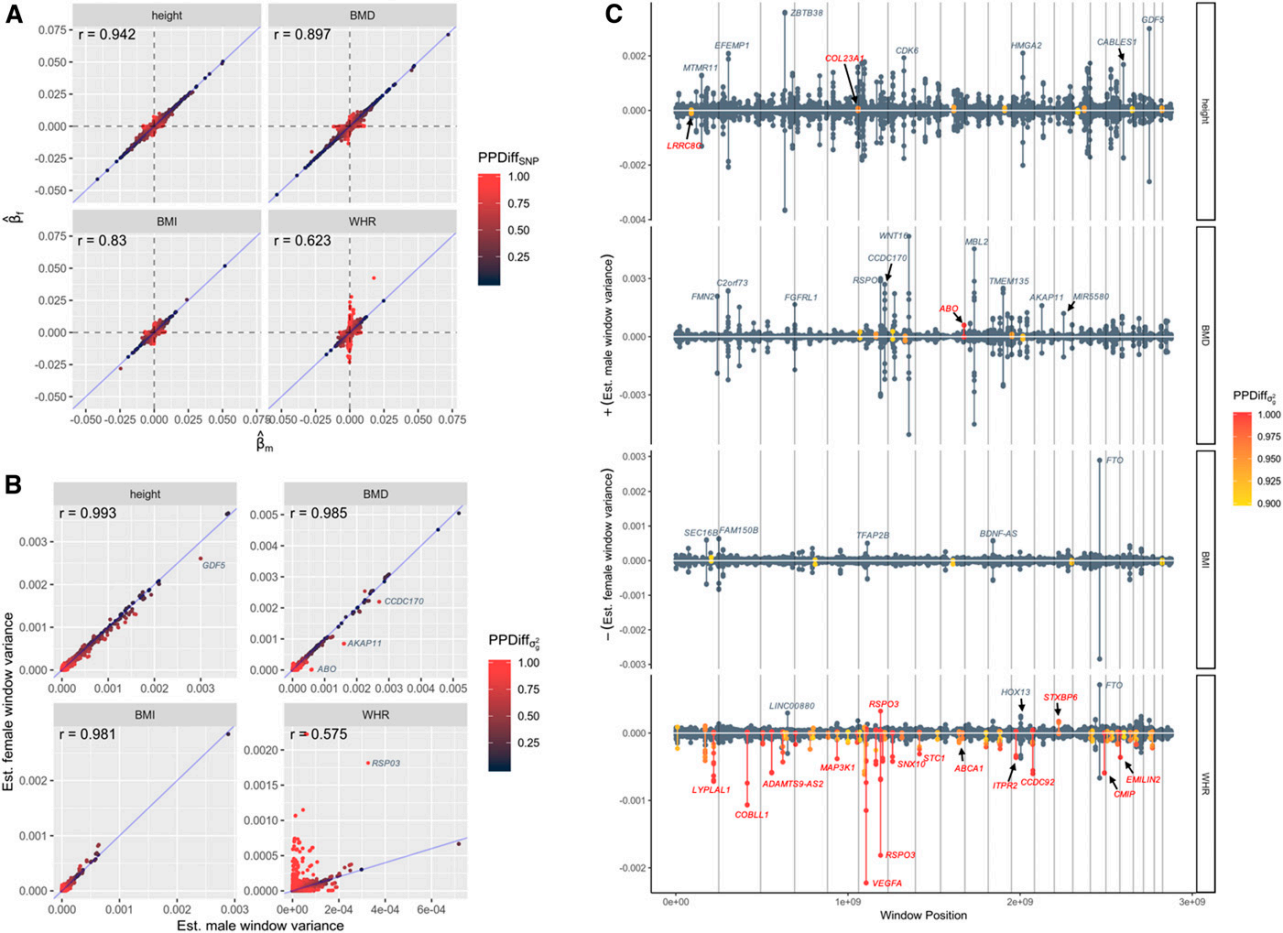
Comparing sex-specific genetic effects. (A) Plot of estimated female SNP effects against estimated male SNP effects for all 607,497 genotyped autosomal SNPs. Points are colored by their posterior probability of sex difference at the level of individual SNPs. (B) Plot of estimated female window variances against estimated male window variances for all 607,497 LD-based windows, with each window j* centered on a different focal SNP *j*. Points are colored by their posterior probability of sex difference at the level of window variances. (C) Miami-like plot depicting location and magnitude of G×S interactions identified through sex-specific window variances. For each trait, showing estimated male window variance above the x-axis and estimated female window variance below the *x*-axis. Vertical lines denote changing chromosomes. A sample of windows is labeled with nearest gene annotation, obtained from Axiom UKB WCSG annotations, release 34. Gray labels indicate nearest genes with relatively large window variances evidently shared across sexes, while red labels indicate nearest genes with detected G×S interactions.

To compare both the magnitude and sign of sex-specific SNP effects, we plotted each ${\hat{\beta }}_{{\text{f}}_{j}}$ against ${\hat{\beta }}_{{\text{m}}_{j}}$ (Figure 4A). The trait was scaled across sexes prior to model fitting; thus, male- and female- specific effects were not constrained to the same scale. In this way, one might expect male-specific SNP effects to uniformly differ from female-specific SNP effects by a multiplicative factor if the variance of the phenotype is different between sexes (sample statistics within each sex are provided within Table S1). Surprisingly, we did not observe evidence of sex-specific SNP effects uniformly differing due to differences in phenotypic scale; for height, BMD, and BMI, as seen in Figure 4A, most large effect SNPs lie near the blue diagonal line. For WHR, we observed largely consistent results from prior studies (Heid *et al.* 2010; Randall *et al.* 2013; Winkler *et al.* 2015): namely the prevalence of numerous SNPs with relatively large effects in females but little to no effect in males. No traits exhibited evidence of any SNPs with (i) high confidence male- and female- specific effects (no SNPs with ${\text{PPM}}_{\text{SNP}}\geq \;0.9$ and ${\text{PPF}}_{\text{SNP}}\geq 0.9$) and (ii) differing signs between sexes.

We then aggregated sex-specific SNP effects within small LD-based regions to estimate sex-specific window variances $\sigma_{g_{\text{m}}{}_{j^{*}}}^{2}$ and $\sigma_{g_{\text{f}}{}_{j^{*}}}^{2}$ and compared the magnitude of each (Figure 4B). Interestingly, for traits such as height, many large effect regions bear slightly larger window variances for males than for females. This was not observed at the single SNP level, suggesting that many regions bearing numerous small effect SNPs produce aggregate effects that are potentially larger (although not reaching a ${\text{PPDiff}}_{\sigma_{g}^{2}}\geq 0.9$ threshold) in males than in females. One example is the *GDF5* locus, previously known to strongly associate with adult height (Sanna *et al.* 2008), where a peak ${\text{PPDiff}}_{\sigma_{g}^{2}}$ signal centered on rs143384 had slightly different estimated sex-specific window variances (${\hat{\sigma }}_{g_{\text{m}}}^{2}=3.0\times 10^{-3}$ and ${\hat{\sigma }}_{g_{\text{f}}}^{2}=2.6\times 10^{-3}$) but weak evidence of a G×S interaction $\left({\text{PPDiff}}_{\sigma_{g}^{2}}=0.544\right)$. For BMD, several large effect regions show suggestive evidence of G×S interactions, including the *AKAP11* locus and the *CCDC170* locus (${\text{PPDiff}}_{\sigma_{g}^{2}}=0.856$ and 0.745, respectively), both previously associated with BMD (Mullin *et al.* 2016, 2017; Correa-Rodríguez *et al.* 2018; Zhu *et al.* 2018).

To make G×S inferences at the level of window variances irrespective of the magnitude of sex-specific effects, we adopted a ${\text{PPDiff}}_{\sigma_{g}^{2}}$ threshold of 0.9, which, in simulations (Figure 3), provided optimal power at an estimated FDR of 0.029 when using a 1-MB target area. For height, a total of eight distinct regions possessed a ${\text{PPDiff}}_{\sigma_{g}^{2}}\geq 0.9,$ two of which possessed a ${\text{PPDiff}}_{\sigma_{g}^{2}}\geq 0.95.$ For BMI, five distinct regions possessed a ${\text{PPDiff}}_{\sigma_{g}^{2}}\geq 0.9,$ with none reaching a more stringent ${\text{PPDiff}}_{\sigma_{g}^{2}}\geq 0.95$ threshold, and none overlapping with two previously suggested BMI G×S SNPs (Locke *et al.* 2015). As seen in Figure 4C, inferred G×S interactions for height and BMI possess relatively small sex-specific window variances; as an example, for height, the window centered on SNP rs1535515 (near *LRRC8C*) had a ${\text{PPDiff}}_{\sigma_{g}^{2}}=0.96,$ whereas ${\hat{\sigma }}_{g_{\text{m}}}^{2}=2.1\times 10^{-5}$ and ${\hat{\sigma }}_{g_{\text{f}}}^{2}=1.1\times 10^{-4}.$ For BMD, seven regions reached a 0.9 ${\text{PPDiff}}_{\sigma_{g}^{2}}$ threshold, while one higher-confidence G×S interaction $\left({\text{PPDiff}}_{\sigma_{g}^{2}}\geq 0.95\right)$ was detected within *ABO*, the gene controlling blood type.

For WHR, roughly 45 distinct genomic regions possessed a ${\text{PPDiff}}_{\sigma_{g}^{2}}\geq 0.9,$ while 34 of these possessed a ${\text{PPDiff}}_{\sigma_{g}^{2}}\geq 0.95.$ We found many previously detected G×S interactions known to associate with WHR or a related trait, WHR adjusted for BMI (WHRadjBMI) (Heid *et al.* 2010; Randall *et al.* 2013; Shungin *et al.* 2015; Winkler *et al.* 2015). These included interactions at *LYPLAL1*, *MAP3K1*, *COBLL1*, *RSPO3*, and *VEGFA*, among others. We also detected numerous novel G×S interactions (Table 1) near physiologically intriguing genes such as the estrogen receptor gene *ESR1* and the ATP binding cassette transporter A1 gene *ABCA1* known to play a role in HDL metabolism $\left({\text{PPDiff}}_{\sigma_{g}^{2}}\geq 0.95\right).$ As seen in Table 1, both novel signals possessed a high-confidence female-specific effect with weak evidence for a male-specific effect (${\text{PPF}}_{\sigma_{g}^{2}}\geq 0.95$; ${\text{PPM}}_{\sigma_{g}^{2}}\leq 0.6$); however, the magnitude of the female-specific effect was relatively small $\left({\hat{\sigma }}_{g_{\text{f}}}^{2}\leq 1.4\times 10^{-4}\right).$ As evident from Table 1, most novel WHR G×S interactions detectable with LBR are those with relatively small sex-specific effects.

**Table 1:** G×S interactions inferred through sex-specific window variances.

Focal SNP*^a^*	trait	${\hat{\sigma }}_{g_{\text{m}}}^{2}$*^b^*	${\hat{\sigma }}_{g_{\text{f}}}^{2}$*^c^*	${\text{PPM}}_{\sigma_{g}^{2}}$	${\text{PPF}}_{\sigma_{g}^{2}}$	${\text{PPDiff}}_{\sigma_{g}^{2}}$	Nearest gene*^d^*	Location	eQTL*^e^*
rs8176719	BMD	0.06	0.00182	1	0.794	1	*ABO*	Exon/frameshift	Yes
rs1535515	height	0.00211	0.0117	0.819	0.999	0.956	*LRRC8C*	Intron	Yes
rs1544926	height	0.00763	0.00035	0.983	0.418	0.955	*COL23A1*	UTR-3	Yes
rs6905288	WHR	0.00567	0.222	0.92	1	1	***VEGFA***	Downstream	
rs72961013	WHR	0.0326	0.181	1	1	1	***RSPO3***	Downstream	
rs1128249	WHR	0.00132	0.107	0.614	1	1	***COBLL1***	Intron	Yes
rs12022722	WHR	0.0008	0.0718	0.49	1	1	***LYPLAL1***	Downstream	Yes
rs1776897	WHR	0.0087	0.0614	0.976	1	0.95	***HMGA1***	Upstream	Yes
rs11057401	WHR	0.00438	0.0603	0.846	1	1	***CCDC92***	Exon/missense	Yes
rs17777180	WHR	0.00031	0.0595	0.291	1	1	***CMIP***	Intron	Yes
rs4607103	WHR	0.00195	0.0592	0.809	1	1	***ADAMTS9-AS2***	Intron	Yes
rs6937293	WHR	0.00457	0.0466	0.839	1	1	***LOC728012***	Downstream	Yes
rs16861373	WHR	0.00066	0.043	0.389	1	0.995	***PLXND1***	Intron	
rs73068463	WHR	0.00068	0.0422	0.461	1	1	***SNX10***	Intron	Yes
rs9376422	WHR	0.00107	0.0418	0.524	1	1	*LOC645434*	Upstream	
rs6867983	WHR	0.00192	0.0382	0.44	1	0.998	***MAP3K1***	Upstream	
rs2171522	WHR	0.00241	0.0365	0.561	1	0.998	***ITPR2***	Downstream	Yes
rs3810068	WHR	0.00026	0.0359	0.174	1	1	*EMILIN2*	Upstream	Yes
rs568890	WHR	0.00129	0.0311	0.809	1	1	***NKX2-6***	Upstream	Yes
rs1332955	WHR	0.00647	0.0294	0.97	1	0.973	***LOC284688***	Downstream	Yes
rs13133548	WHR	0.00019	0.024	0.175	0.969	0.956	***FAM13A***	Intron	Yes
rs11263641	WHR	0.00207	0.0234	0.723	1	0.991	*MYEOV*	Downstream	Yes
rs2800999	WHR	0.00201	0.0222	0.691	1	0.979	*TSHZ2*	Intron	
rs2244506	WHR	0.00101	0.0207	0.453	0.998	0.985	*MIR5694*	Downstream	
rs7259285	WHR	0.00182	0.0171	0.767	1	0.989	*HAUS8*	Downstream	Yes
rs4450871	WHR	0.00002	0.0168	0.027	1	1	*CYTL1*	Downstream	
rs4080890	WHR	0.00153	0.0163	0.594	0.999	0.975	***KCNJ2***	Downstream	
rs4684859	WHR	0.00039	0.0157	0.33	0.998	0.994	***PPARG***	Downstream	
rs7704120	WHR	0.00049	0.0137	0.476	0.998	0.991	*STC2*	Downstream	
rs10991417	WHR	0.00048	0.0123	0.339	0.986	0.966	*ABCA1*	Intron	Yes
rs12454712	WHR	0.00087	0.0102	0.36	0.996	0.965	***BCL2***	Intron	Yes
rs62070804	WHR	0.00004	0.00887	0.052	0.969	0.961	*ABHD15*	Exon/missense	Yes
rs10760322	WHR	0.00027	0.00812	0.282	0.986	0.968	*LHX2*	Downstream	
rs1361024	WHR	0.00022	0.0076	0.203	0.982	0.962	*ESR1*	Intron	
rs1358503	WHR	0.00021	0.00716	0.309	0.989	0.966	*SEMA3C*	Upstream	Yes
rs13156948	WHR	0.00016	0.0066	0.079	0.97	0.957	*IRX1*	Downstream	
rs12432376	WHR	0.0174	0.00074	1	0.552	0.994	*STXBP6*	Upstream	

Listed are loci with at least 0.95 posterior probability that sex-specific window variances differ. The table is sorted first by trait, then by magnitude of the female-specific window variance. Results are filtered such that each window listed consisted of a distinct set of SNPs. A full list of all G×S signals at a ${\text{PPDiff}}_{\sigma_{g}^{2}}$≥ 0.90 threshold is provided in Table S4.

a Focal SNP is defined as the center SNP *j* in window j*.

b The proportion of variance explained by male-specific SNP effects, expressed as a percentage.

c The proportion of variance explained by female-specific SNP effects, expressed as a percentage.

d Nearest gene and location identified through Axiom UKB WCSG annotations, release 34. The gene/locus is bold if it has been previously detected as a G×S interaction for WHR or WHR adjusted for BMI (Heid *et al.* 2010; Randall *et al.* 2013; Shungin *et al.* 2015; Winkler *et al.* 2015).

e If “yes,” the focal SNP is significantly associated with gene expression in at least one tissue, according to GTEx V7

Additionally, we utilized a traditional SMR approach (see Supplemental Methods) for the discovery of G×S interactions among traits to compare *P value*-diff signals to ${\text{PPDiff}}_{\sigma_{g}^{2}}$ signals (Figure S4). At *P value*-diff ≤ 5 × 10^−8^, there were no genome-wide significant G×S-interacting SNPs for height, one significant SNP for BMI near a window with ${\text{PPDiff}}_{\sigma_{g}^{2}}\geq 0.9,$ and one significant peak within *ABO* for BMD (the same signal detected using ${\text{PPDiff}}_{\sigma_{g}^{2}}$). Regions with a ${\text{PPDiff}}_{\sigma_{g}^{2}}\geq 0.9$ generally coincided with at least nominally significant *P value*-diff signals; for height and BMD, regions with ${\text{PPDiff}}_{\sigma_{g}^{2}}\geq 0.9$ also possessed a peak SNP with *P value*-diff ≤ 0.01. For BMI, ${\text{PPDiff}}_{\sigma_{g}^{2}}\geq 0.9$ signals possessed a peak SNP of *P value*-diff ≤ 0.1. This, together with the fact that novel G×S interactions found using LBR possess relatively small sex-specific effects, suggests that LBR may be detecting G×S interactions that are otherwise missed due to low power. Lastly, for WHR, most of the high-confidence ${\text{PPDiff}}_{\sigma_{g}^{2}}\geq 0.9$ signals coincided with clear and obvious *P value*-diff peaks.

### Inferred G×S interactions were enriched in tissue-specific eQTL

As seen previously, many G×S interactions inferred using LBR have exceedingly small sex-specific effects. To further investigate whether G×S detections using the ${\text{PPDiff}}_{\sigma_{g}^{2}}$metric may be functionally relevant, we inferred whether such signals are enriched in eQTL identified from GTEx. Specifically, using a hyper-geometric test we asked whether ${\text{PPDiff}}_{\sigma_{g}^{2}}$-selected focal SNPs (SNP *j* within window j*) were enriched in eQTL, then compared to eQTL enrichment from *P value*-diff-selected SNPs as a function of the number of SNPs selected (Figure S5). ${\text{PPDiff}}_{\sigma_{g}^{2}}$-selected focal SNPs showed consistently higher eQTL enrichment than *P value*-diff-selected SNPs for all traits except WHR. For instance, at ${\text{PPDiff}}_{\sigma_{g}^{2}}\geq 0.9,$ the total number of windows (focal SNPs) selected was 36, 264, 34, and 13, for height, WHR, BMD, and BMI, respectively. With these selections, eQTL enrichment *P*-values were 2.39 × 10^−4^, 1.52 × 10^−12^, 2.01 × 10^−12^, and 8.33 × 10^−4^, for height, WHR, BMD, and BMI, respectively. When selecting the same number of SNPs using *P value*-diff, enrichment *P*-values were 2.25 × 10^−2^, 1.56 × 10^−28^, 5.54 × 10^−8^, 1.93 × 10^−1^, for height, WHR, BMD, and BMI, respectively.

**Figure 5:**
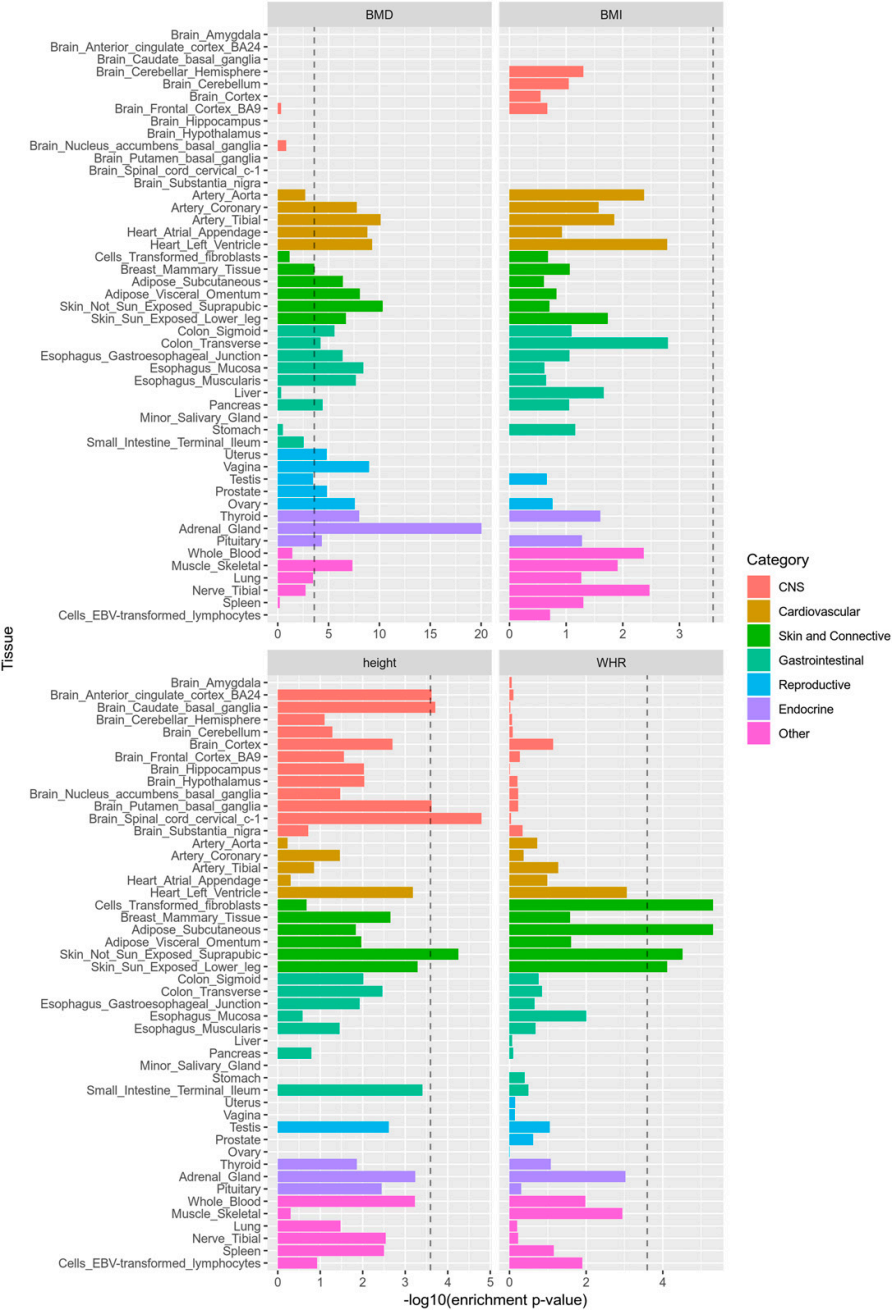
Evidence that LBR-identified G×S interactions are enriched in tissue-specific eQTL. Plotted on the *x*-axis is the *P*-value obtained from a hyper-geometic test providing evidence that focal SNPs selected using ${\text{PPDiff}}_{\sigma_{g}^{2}}\geq 0.9$ are enriched in tissue-specific eQTL. The dashed line represents a Bonferroni corrected significance threshold of 2.6 × 10^−4^.

To provide more information about how genetic regions bearing G×S interactions may impact gene expression in specific tissues, we determined whether focal SNPs at ${\text{PPDiff}}_{\sigma_{g}^{2}}\geq 0.9$ are enriched in tissue-specific eQTL (Figure 5). For height, BMD, and WHR, such SNPs showed significant eQTL enrichment in at least one tissue, using a conservative Bonferroni corrected enrichment *P*-value of 2.6 × 10^−4^ (correcting for 192 tests in total; 48 tissues and four traits). Interestingly, BMD G×S signals are very strongly enriched in eQTL with associated eGenes (including *ABO* and *CYP3A5*) expressed in the adrenal gland, among other tissues. For height, we observed small enrichment *P*-values across many tissues since G×S focal SNPs are enriched in eQTL with associated eGenes (including *LOC101927975* and *CNDP2*) expressed across many tissues. Lastly, for WHR, we observed G×S detections to be heavily enriched in eQTL with associated eGenes expressed in fibroblast, adipose, and skin tissues.

## Discussion

We have investigated the degree to which sex-specific genetic architectures differ at local regions, using large biobank data (N ∼119,000 males and ∼140,000 females) and Bayesian multiple regression techniques that estimate sex-specific marker effects accounting for local LD patterns. The flexibility of the Bayesian approach enables multi-resolution inference of sex-specific effects, from individual SNP effects to window-variances that aggregate SNP effects within chromosome segments. These inferences can all be drawn using the results of the same model fit (Equation 1), but different postprocessing of samples of SNP effects from the posterior distribution.

The Bayesian multiple regression technique performed in this study, along with estimation of window variances, was largely inspired by Fernando *et al.* (2017). In that study, windows were defined using disjoint, fixed intervals. In contrast, for each SNP, we define a window based on local LD patterns, resulting in heavily overlapping, dynamically sized windows. The methods presented here also bear resemblance to those of Vilhjálmsson *et al.* (2015), which utilized point-normal priors to estimate human SNP effects after accounting for local LD patterns. In that study, posterior means of SNP effects were estimated for the purposes of prediction, whereas, in this study, we derive the full posterior distribution numerically, allowing for inference of non-null SNP effects and window variances.

Through simulations, we showed that LBR provides superior power and precision to detect causal variants and those specifically bearing G×S interactions. We rationalize improvements in power upon traditional SMR methods by noting that the magnitude of a typical causal variant or G×S interaction is exceedingly small, and can elude hypothesis testing, due partly to the burden of multiple test correction. We also note that the resolution (peak size) in SMR signals is relatively large when using large sample sizes (due to not fully accounting for local LD patterns). To overcome this problem, we provided evidence that LBR methods—either by estimating conditional (accounting for local LD) marker effects or by aggregating conditional marker effects within relatively small regions—can achieve improved resolution when working with large sample sizes such as biobank-level data.

When using LBR to analyze real human traits, we have provided credence to our posterior probability-based discoveries by determining that LBR-detected G×S interactions are generally more enriched in eQTL than SMR-detected interactions. For BMD, we provided new evidence that sex-specific effects differ within *ABO*, and that G×S interactions are highly enriched in adrenal gland-specific eQTL. This encourages the hypothesis that some G×S are eQTL that may modulate gene expression in the adrenal gland, with gene function dependent on the level of sex hormones. This was also an intriguing finding given that ABO blood groups have been known to associate with osteoporosis and osteoporosis severity (Choi and Pai 2004; Lu and Li 2011). For WHR, we detected previously known, large-magnitude G×S interactions that were discovered using WHR or WHRadjBMI (Heid *et al.* 2010; Randall *et al.* 2013; Shungin *et al.* 2015; Winkler *et al.* 2015), but additionally discovered novel, small magnitude G×S interactions near such genes as *ESR1* and *ABCA1*. In a previous work analyzing WHRadjBMI, *ABCA1* showed a significant female-specific genetic effect only; however, the test for G×S interaction failed to reach significance (Shungin *et al.* 2015).

For traits like height and BMI, large effect loci are estimated to have very similar effects between males and females, and loci with evidence of G×S interactions were those possessing relatively small sex-specific effects. As seen in Figure 4B, many relatively large window variances for height are estimated to be slightly higher for males than for females, albeit not reaching a ${\text{PPDiff}}_{\sigma_{g}^{2}}\geq 0.9$ threshold. This is consistent with the fact that the global genomic variance for height was estimated to be higher in males than in females in a previous study using the interim release of the UK Biobank (Rawlik *et al.* 2016). Similarly, the same prior study estimated the global genomic variance of BMI to be higher in females than in males, and we observe, if anything, evidence of sex-specific window variances leading to the same conclusion. These observations may potentially indicate that relatively large causal variants have slightly different sex-specific effects for traits like height and BMI; however, if that is the case, we are still underpowered to confidently detect such interactions.

It is important to acknowledge that, while the methods presented here appear useful to decipher sex-specific genetic architectures from large human samples, additional work will be required to determine how these techniques may infer heterogeneous genetic effects in other contexts (other types of gene-by-covariate interactions), or when using different sample sizes or samples from different populations. With large sample sizes, the increased power and flexibility of LBR comes at the cost of a significantly larger computational burden than that involved in the traditional SMR approach; however, working with large datasets can be made manageable by adjusting the size of each fitted segment (Figure 1) and parallel processing the fitting of each segment. Alternatively, LBR may be used as a follow up to traditional SMR tests, using preselected regions of interest. Another limitation inherent to aggregating SNP effects using window variances is that the sign of the effect is lost. In this way, when inferring G×S interactions through window variance differences, we cannot comment on whether sex-specific effects had the same sign or differing signs.

To conclude, we have demonstrated the powerful and flexible use of local Bayesian regressions for GWA to infer sex-specific genetic effects and G×S interactions using the UK Biobank. This was done largely by showing various means to utilize estimates of conditional (accounting for local LD), sex-specific SNP marker effects for GWA even when causal variants are not on the SNP panel for analysis. We anticipate that many more traits will be analyzed with this method to increasingly learn more about what is contributing to differences between males and females in human populations.
